# Plasma Asymmetric Dimethylarginine and Adverse Events in Patients with Atrial Fibrillation Referred for Coronary Angiogram

**DOI:** 10.1371/journal.pone.0071675

**Published:** 2013-08-07

**Authors:** Tze-Fan Chao, Tse-Min Lu, Yenn-Jiang Lin, Hsuan-Ming Tsao, Shih-Lin Chang, Li-Wei Lo, Yu-Feng Hu, Ta-Chuan Tuan, Ming-Hsiung Hsieh, Shih-Ann Chen

**Affiliations:** 1 Division of Cardiology, Department of Medicine, Taipei Veterans General Hospital, Taipei, Taiwan; 2 Institute of Clinical Medicine, and Cardiovascular Research Center, National Yang-Ming University, Taipei, Taiwan; 3 Division of Cardiology, National Yang Ming University Hospital, I-Lan, Taiwan; 4 Division of Cardiology, Department of Medicine, Wan-Fang Hospital, Taipei Medical University, Taipei, Taiwan; Maastricht University, Netherlands

## Abstract

**Objectives:**

Elevated plasma levels of asymmetric dimethylarginine (ADMA) have been reported to be associated with endothelial dysfunction, inflammation, and oxidative stress in multiple cardiovascular diseases. This study aimed to investigate whether ADMA was a predictor of clinical outcomes in atrial fibrillation (AF).

**Methods and Results:**

From 2006-2009, 990 individuals were referred to our institution for coronary angiography. Among these patients, 141 subjects with a diagnosis of AF, including 52 paroxysmal AF (PAF) and 89 non-paroxysmal AF (non-PAF) patients, were identified as the study population. Plasma ADMA levels were measured. An adverse event was defined as the occurrence of ischemic stroke or cardiovascular death. The ADMA levels were higher in AF than non-AF patients (0.50±0.13 versus 0.45±0.07 µmol/L; p<0.001). Besides, non-PAF patients had higher ADMA levels than PAF patients (0.52±0.15 versus 0.48±0.08 µmol/L; p<0.001). During the follow-up of 30.7±14.4 months, 21 patients (14.9%) experienced adverse events, including cardiovascular death in 7 patients and ischemic stroke in 14. ADMA level, CHA_2_DS_2_-VASc score, and left atrial diameter were independent predictors of adverse events in the multivariate analysis. At a cutoff-value of 0.55 µmol/L, the Kaplan-Meier survival analysis showed that patients with a high ADMA level had a higher event rate during the follow-up period.

**Conclusions:**

A higher level of ADMA was a risk factor of adverse events in AF patients, which was independent from the CHA_2_DS_2_-VASc score. It deserves to further study whether ADMA could potentially refine the clinical risk stratification in AF.

## Background

Atrial fibrillation (AF) is the most common sustained cardiac arrhythmia, with an estimated prevalence rate ranging from 0.4% to 1% in the general population.[1], [2] Its incidence is projected to increase continuously over the next few decades, and therefore AF has been referred to as a “non-contagious epidemic”.[3] It is associated with a 5- to 6-fold increase in the risk of cerebrovascular events and is the leading cause of ischemic strokes.[4]

Asymmetric dimethylarginine (ADMA), an analogue of L-arginine, is a naturally occurring product of metabolism found in human circulation. Elevated levels of ADMA can inhibit nitric oxide synthase (NOS) and lead to endothelial dysfunction, inflammation, and oxidative stress in multiple cardiovascular diseases.[5], [6] High plasma ADMA levels have been reported to be an independent risk factor for adverse cardiovascular events and mortality in patients with coronary artery disease (CAD),[7] chronic kidney disease,[8] and after acute ischemic stroke.[9] Recently, several studies have shown that ADMA levels are high in AF patients,[10], [11] and are associated with a higher recurrence rate after cardioversion or catheter ablation for AF.[12], [13] However, the data regarding the relationship between ADMA and adverse events in AF patients are limited. Thus, the goal of the present study was to investigate whether the plasma levels of ADMA are a predictor of poor clinical outcomes in patients with AF.

## Methods

### Ethics Statement

The study protocol was approved by the Institutional Review Board at Taipei Veterans General Hospital and informed written consent was obtained from each participant in accordance with the ethical guidelines of the Declaration of Helsinki.

### Study Population

From July 2006 to June 2009, we enrolled 990 individuals who were referred to our institution for coronary angiography. Among these patients, 141 subjects with a diagnosis of AF, including 52 paroxysmal AF (PAF) and 89 non-paroxysmal AF (non-PAF) patients, were identified as the study population.[14], [15] Blood samples were collected before diagnostic coronary angiography, which was then performed using a standard procedure. The CHA_2_DS_2_-VASc score was calculated for each AF patient based on a point system in which 2 points are assigned for a history of stroke or transient ischemic attack, or an age ≥ 75 years, and 1 point is assigned for each of the following factors: age 65–74 years, a history of hypertension, diabetes, recent cardiac failure, vascular disease (CAD, myocardial infarction, complex aortic plaque, or peripheral artery disease), and female sex.[16] Percutaneous coronary intervention or coronary artery bypass surgery was recommended for patients if significant CAD was diagnosed, which was defined as the presence of > 50% stenosis in at least one major coronary artery according to the results of coronary angiography.

### Laboratory Measurement of ADMA

Before performing coronary angiography, blood samples were collected into tubes containing ethylenediaminetetraacetic (EDTA) as an anticoagulant and were centrifuged at 3000 rpm for 10 minutes at 4°C immediately after collection. Plasma samples were kept frozen at –70°C until analysis. Plasma ADMA concentrations were determined by high performance liquid chromatography using precolumn derivatization with o-phthaldialdehyde as previously described.[17] The recovery rate for ADMA was > 90%, and the within-assay and between-assay variation coefficients were not greater than 7% and 8%, respectively.

### Definitions of the Clinical Endpoints and Follow-up

Patients were prospectively followed by the office visit monthly or by telephone contact and chart review for the occurrence of adverse events, defined as ischemic stroke or cardiovascular death. Ischemic stroke was defined as a focal neurological deficit of sudden onset as diagnosed by a neurologist, lasting > 24 hours, and caused by ischemia. Imaging studies of brain, including computed tomography scan or magnetic resonance imaging, were performed for each patient to exclude the presence of intra-cranial hemorrhage and confirm the diagnosis. Cardiovascular death was diagnosed as any death with definite cardiovascular cause or any death that was not clearly attributed to a non-cardiovascular cause.

### Statistical Analysis

The data are presented as mean values and standard deviations for normally distributed continuous variables, medians and interquartile ranges for skewed data, and proportions for categorical variables. The differences between normally distributed continuous values were assessed using an unpaired two-tailed t-test or one-way analysis of variance (ANOVA) with post-hoc Bonferroni test. The Mann-Whitney rank-sum test was used for the analysis of skewed variables, and the differences between nominal variables were compared with a chi-squared test. A Cox regression analysis was used to identify the factors associated with adverse events. The optimal cut-off value of the ADMA levels in the prediction of adverse events was identified using the receiver operating characteristic (ROC) curve. The event-free survival curve was plotted using the Kaplan-Meier method with statistical significance examined by the log-rank test. All statistical significances were set at p<0.05 and all statistical analyses were carried out by SPSS 17.0 (SPSS Inc. USA).

## Results

### Baseline Characteristics and ADMA Levels

**Figure 1:**
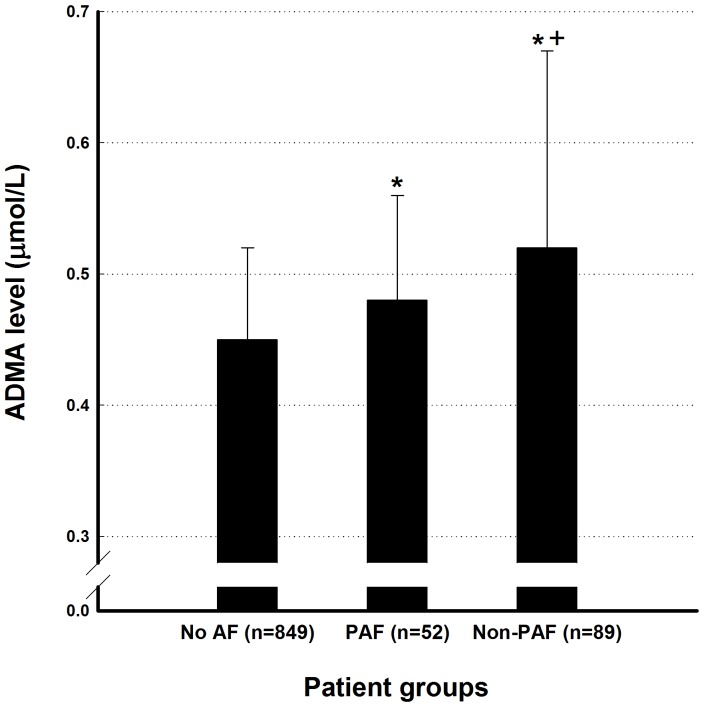
ADMA levels in AF and non-AF patients. The ADMA levels were higher in AF patients compared to non-AF patients. In addition, non-PAF patients had higher levels of ADMA than PAF patients. ADMA  =  asymmetric dimethylarginine; AF  =  atrial fibrillation; PAF  =  paroxysmal atrial fibrillation. *P value <0.05, PAF or non-PAF versus no AF. ^+^P value <0.05, Non-PAF versus PAF.

The mean age of the study population (AF patients, n = 141) was 79.3±4.2 years (range, 58–89 years), and 90.8% of them were male. Hypertension was the most common comorbidity, which was present in 81.6% of all patients. The mean left atrial (LA) diameter and left ventricular ejection fraction (LVEF) were 39.1±7.1 mm and 49.8±12.2%, respectively. Significant CAD was present in 89 patients (63.1%), and most of them (87 patients) received coronary interventional procedures. The ADMA levels were higher in AF patients than in non-AF patients (0.50±0.13 versus 0.45±0.07 µmol/L; p<0.001). In addition, non-PAF patients had higher ADMA levels than PAF patients (0.52±0.15 versus 0.48±0.08 µmol/L; p<0.001)(Figure 1). Besides, plasma ADMA levels were significantly higher in patients with significant CAD (≥50% stenosis, n = 639) than those with insignificant CAD (20–50% stenosis, n = 282) and normal coronary artery (<20% stenosis, n = 69) (0.47±0.10 versus 0.44±0.09 versus 0.42±0.08 μmol/L, p<0.001).

### Predictors of Adverse Events

During the follow-up period of 30.7±14.4 months, 21 patients (14.9%) experienced adverse events, including cardiovascular death in 7 patients and ischemic stroke in 14. The baseline characteristics of patients with and without events are shown in Table 1. Patients with adverse events had lower body mass index (BMI), larger LA diameters, lower LVEF, higher ADMA levels, and higher CHA_2_DS_2_-VASc scores than patients without events. Significant predictors of adverse events based on the univariate Cox regression analysis are shown in Table 2. The ADMA level, CHA_2_DS_2_-VASc score, and LA diameter remained as independent predictors of adverse events in the multivariate model (Table 3). When we only adjusted ADMA and history of vascular disease in the regression model, the level of ADMA was still a significant predictor of adverse events with an adjusted hazard ratio of 1.456 per 0.1 µmol/L increment (95% confidence interval  =  1.182–1.795, p<0.001).

**Table 1:** Baseline characteristics of the patients with and without adverse events.

Variable	With events (n = 21)	Without events (n = 120)	P value
Age, years	81.5±3.8	79.0±4.1	0.010
Gender (male)	95.2%	90.0%	0.391
Smoking	14.3%	12.5%	0.525
Medical history (components of the CHA_2_DS_2_-VASc scoring system)			
Congestive heart failure	66.7%	31.7%	0.002
Hypertension	76.2%	82.5%	0.337
Diabetes mellitus	42.9%	36.7%	0.589
Previous stroke/TIA	19.0%	5.0%	0.042
Vascular disease*	100%	72.5%	0.002
Significant CAD diagnosed by angiography	61.9%	63.3%	0.900
Coronary interventional procedures			
Undergoing PCI	52.4%	55.8%	0.769
Undergoing bypass surgery	4.8%	6.7%	0.601
Use of anti-platelet agents	100%	100%	1
Use of warfarin	19.0%	20.0%	0.593
Body mass index (kg/m^2^)	23.2±4.2	25.3±3.4	0.016
Left atrial diameter, mm	42.4±5.8	38.6±7.1	0.002
LVEF, %	44.7±13.6	50.7±11.7	0.038
AF type (paroxysmal AF)	38.1%	36.7%	0.90
CHA_2_DS_2_-VASc score, median (inter-quartile range)	5 (4–6)	4 (4–5)	0.010
ADMA level (µmol/L)	0.62±0.19	0.48±0.11	0.004

ADMA  =  asymmetric dimethylarginine; AF  =  atrial fibrillation; CAD  =  coronary artery disease; LVEF  =  left ventricular ejection fraction; PCI  =  percutaneous coronary intervention; TIA  =  transient ischemic attack.

* In addition to history of myocardial infarction, complex aortic plaque, or peripheral artery disease, patients with significant CAD diagnosed by coronary angiography at the enrollment were also classified as having vascular disease, and assigned 1 point for calculating the CHA_2_DS_2_-VASc score.

**Table 2:** Univariate Cox regression analysis for predictors of adverse events.

Variables	Hazard ratio	95% CI	P value
Body mass index (per kg/m^2^)	0.847	0.744–0.965	0.012
Left atrial diameter (per mm)	1.058	1.010–1.109	0.018
LVEF (per percent)	0.964	0.931–0.997	0.035
CHA_2_DS_2_-VASc score	1.936	1.282–2.923	0.002
ADMA level (per 0.1 µmol/L)	1.546	1.257–1.902	<0.001

ADMA  =  asymmetric dimethylarginine; LVEF  =  left ventricular ejection fraction.

**Table 3:** Multivariate Cox regression analysis for predictors of adverse events.

Variables	Hazard ratio	95% CI	P value
Body mass index (per kg/m^2^)	0.931	0.821–1.056	0.267
Left atrial diameter (per mm)	1.081	1.022–1.143	0.007
LVEF (per percent)	0.987	0.945–1.030	0.540
CHA_2_DS_2_-VASc score	1.599	1.047–2.440	0.030
ADMA level (per 0.1 µmol/L)	1.364	1.072–1.736	0.012

ADMA  =  asymmetric dimethylarginine; LVEF  =  left ventricular ejection fraction.

**Figure 2:**
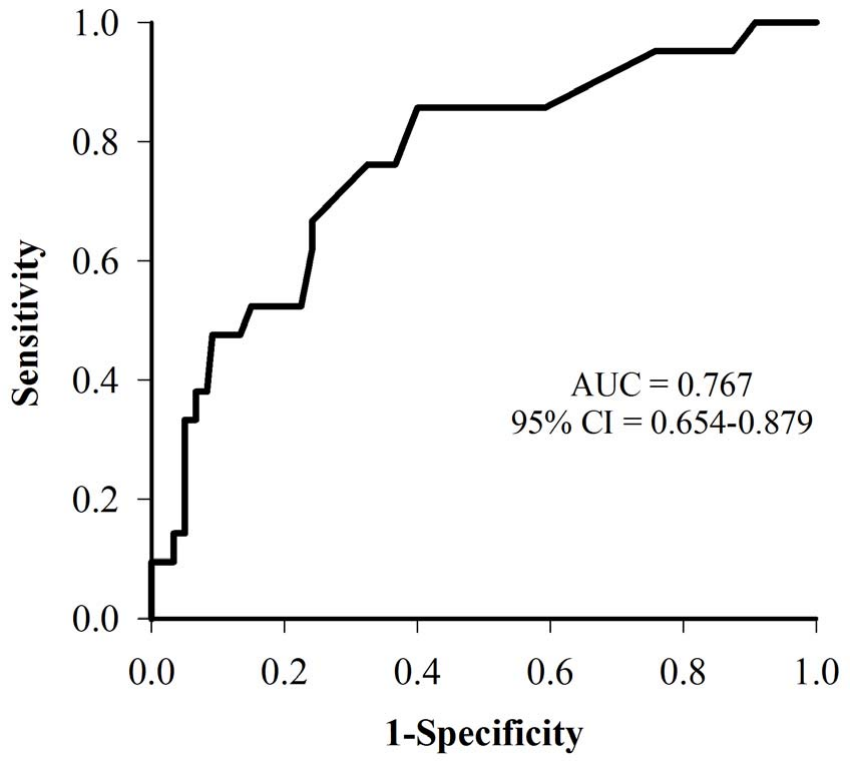
ROC curve for the ADMA levels in predicting adverse events. The area under the curve for ADMA levels in predicting adverse events was 0.767 (95% confidence interval  =  0.654–0.879). ADMA  =  asymmetric dimethylarginine; ROC curve  =  receiver-operator characteristics curve.

**Figure 3:**
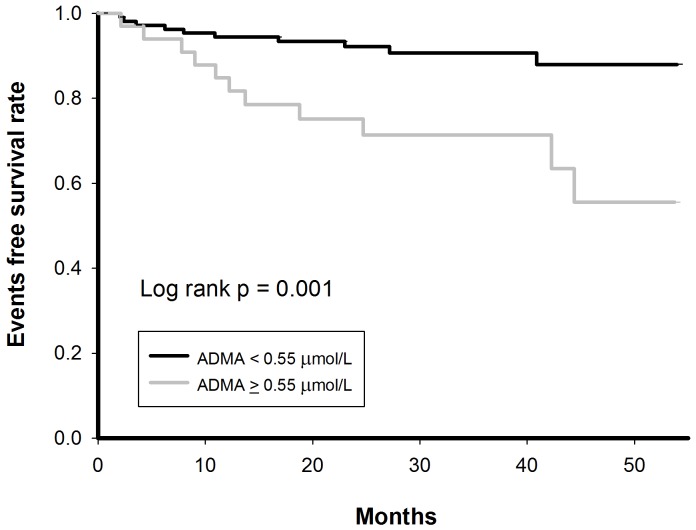
Event-free survival curve for patients with different ADMA levels. Kaplan-Meier survival analysis showed that the patients with an ADMA level ≥ 0.55 µmol/L were associated with a higher event rate compared to patients with an ADMA level <0.55 µmol/L (33.3% versus 9.3%, p = 0.001). ADMA  =  asymmetric dimethylarginine.

Figure 2 shows the ROC curve for predicting events based on the ADMA levels. At a cutoff value of 0.55 µmol/L identified by the ROC curve, the Kaplan-Meier survival analysis showed that patients with an ADMA level > 0.55 µmol/L (sensitivity  =  57.1%; specificity  =  81.7%) were associated with a higher event rate compared to patients with an ADMA level <0.55 µmol/L (33.3% versus 9.3%, p = 0.001) during the follow-up period (Figure 3).

## Discussion

### Main Findings

The main findings of the present study were as follows: (1) ADMA levels were higher in AF patients than non-AF patients. Moreover, non-PAF patients had higher ADMA levels than PAF patients. (2) A higher level of ADMA was a risk factor of adverse events in AF patients, which was independent from the CHA_2_DS_2_-VASc score.

### Increased Level of ADMA in AF – Is it a cause or a result?

Several previous studies have investigated the association between the levels of ADMA and AF.[10], [11] Cengel et al. demonstrated that the plasma concentration of ADMA was significantly increased in AF patients compared to that of healthy controls.[10] This finding was further confirmed in the study performed by Goette et al. which enrolled 35 AF and 25 non-AF subjects, and demonstrated that ADMA levels were higher in AF patients compared to sinus rhythm patients.[11] However, the question that remains is if the increased ADMA levels are a cause or result in AF? Liu et al. demonstrated that AF could increase the expression of protein arginine methyltransferease-1 (PRMT-1), which would lead to an increased ADMA concentration.[18], [19] The previous study also showed that ADMA levels decreased to normal values within 24 hours after AF was terminated by electrical cardioversion. These observations suggest that the higher ADMA levels may be a consequence of AF.[11] On the other hand, ADMA was recently demonstrated to have cytokine-like properties by activating polymorphonuclear neutrophils and enhancing the release of myeloperoxidase, which may increase systemic inflammatory burden and play an important role in the pathogenesis of AF.[20] In addition, increased serum ADMA concentrations were reported to be associated with a higher rate of AF recurrence after catheter ablation or electrical cardioversion in patients with persistent AF.[13], [21] These findings may imply that ADMA itself might participate in the process of atrial remodeling and result in the occurrence of AF.

Based on the previous findings described above, we hypothesized that the increased ADMA levels may predispose patients to AF, which could further increase the concentration of ADMA and form a vicious cycle. The results of our study support this hypothesis. The observation of higher ADMA levels in PAF patients compared to non-AF patients suggest that ADMA is a cause of AF, and the further increased ADMA levels in non-PAF patients imply that sustained AF could result in an increased concentration of ADMA.

### ADMA and Adverse Events in AF

AF patients constitute a high-risk population for cardiovascular events and mortality, and the risk varies according to several demographic and clinical characteristics, such as heart failure, hypertension and diabetes mellitus. Recently, a newly developed scoring system, the CHA_2_DS_2_-VASc score, which extends the CHADS_2_ scheme by considering additional stroke risk factors (vascular diseases and female gender), was recommended for guiding antithrombotic therapies for AF patients.[15], [16] In addition to the clinical risk score, several biomarkers, including cardiac troponin T, interleukin-6, and plasma von Willebrand factor, were reported to be useful in predicting adverse events in AF.[22], [23] In the present study, we demonstrated that an increased level of ADMA may be a risk factor of poor prognosis for AF patients even after adjustment with the CHA_2_DS_2_-VASc score. This finding is consistent with a previous report showing that increased ADMA levels were present in patients with acute cardio-embolic infarction compared to controls.[24]

What is the mechanism behind the usefulness of ADMA in predicting adverse events in AF? By inhibiting the formation of endothelial nitric oxide (NO), ADMA accumulation in AF may result in endothelial dysfunction in LA and the LA appendage, which may lead to thrombus formation and subsequent thromboembolic events.[18] In addition to being a vasodilator, NO also inhibits platelet adhesion and aggregation, attenuates monocyte infiltration, suppresses myointimal hyperplasia and reduces oxidative stress.[25] The decrease in NO availability could therefore enhance atherosclerosis and local inflammation of the vessel wall, which may play an important role in plaque rupture, thereby leading to stroke or myocardial infarction.[26] Furthermore, previous studies have also shown that the infusion of ADMA in healthy volunteers would increase arterial stiffness and decrease cerebral perfusion.[27], [28] Taken together, these evidences suggest that ADMA might be the potential link between AF, increased oxidative stress, endothelial dysfunction, thrombogenesis and atherosclerosis, and could therefore represent a risk factor of adverse events in AF patients.

Another interesting finding of the present study was that patients with a higher BMI had a lower event rate. The potentially protective effect of increased BMI in AF has been reported previously and termed the “obesity paradox”.[29], [30] The possible explanation is that there were more patients with heart failure in the group with events, and “cardiac cachexia” may predispose patients to a poor prognosis. The speculation is further supported by the finding that BMI was not a significant predictor of adverse events after the adjustment for other factors, including LVEF, LA diameter and the CHA_2_DS_2_-VASc score, which includes heart failure in the scoring scheme.

### Comparison with Previous Studies

Compared to previous studies investigating AF, ADMA and cardiovascular risk, the patient number in our study was larger than that of previous ones. We specifically analyzed the ADMA levels in PAF and non-PAF patients, which could provide a further evidence to suggest that ADMA may involve in the process of AF progression. Besides, the relationship between ADMA and adverse events in pure AF patients, especially when considering CHA_2_DS_2_-VASc score, has not been explored before. To the best of our knowledge, the present study is the first one to demonstrate the significant link between ADMA and a poor prognosis in AF.

### Study Limitations

There were several limitations of the present study. First, the study patients were enrolled from a population undergoing diagnostic coronary angiography and had a high prevalence of CAD. Besides, a high percentage of study patients (90.8%) were male. Whether the results presented here could be applied to other populations remained uncertain. Second, oral anticoagulants were underused for stroke prevention in our cohort due to the concern of high risk of bleeding (old age and concurrent use of anti-platelet agents). However, this is a common problem in managing AF patients in daily clinical practice.[31], [32] Besides, the use of warfarin was not a significant factor associated with adverse events in the Cox regression analysis, and therefore it may not confound the results demonstrating the usefulness of ADMA in predicting adverse events. Lastly, there were no low-risk patients with a CHA_2_DS_2_-VASc score of 0 or 1 enrolled in the present study. Therefore, we were not able to analyze whether the measurement of ADMA levels could be helpful in identifying patients at risk among these low-risk patients. The accumulated evidence shows that CHA_2_DS_2_-VASc score is better than the CHADS_2_ score in identifying patients who develop stroke and thromboembolism,[33], [34] and it has expanded the indication for oral anticoagulants in AF patients.[35] It deserves a large-scale and prospective trial to confirm our findings and investigate whether ADMA could potentially refine the clinical risk stratification and improve decision-making for thromboprophylaxis in patients with AF.

## Conclusions

ADMA levels were found to be higher in AF than non-AF patients. Furthermore, non-PAF patients had higher ADMA levels than PAF patients. A higher level of ADMA was a risk factor of adverse events in AF patients, which was independent from the CHA_2_DS_2_-VASc score in this high-risk and male predominant population with a high prevalence of CAD. The potential role of plasma ADMA levels in predicting adverse events in addition to the CHA_2_DS_2_-VASc score deserves further investigation.
